# Innovative Therapeutic Strategies in Alzheimer’s Disease: A Synergistic Approach to Neurodegenerative Disorders

**DOI:** 10.3390/ph17060741

**Published:** 2024-06-06

**Authors:** Sarfaraz K. Niazi, Matthias Magoola, Zamara Mariam

**Affiliations:** 1 College of Pharmacy, University of Illinois, Chicago, IL 60012, USA; 2 DEI Biopharma, Kampala P.O. Box 35854, Uganda; dei@deigroupinternational.com; 3 Centre for Health and Life Sciences, Coventry University, Coventry CV1 5FB, UK

**Keywords:** neurodegenerative diseases, Alzheimer’s disease, mRNA vaccine, antibody targeting, chemical targeting, blood–brain barrier, nanobodies

## Abstract

Alzheimer’s disease (AD) remains a significant challenge in the field of neurodegenerative disorders, even nearly a century after its discovery, due to the elusive nature of its causes. The development of drugs that target multiple aspects of the disease has emerged as a promising strategy to address the complexities of AD and related conditions. The immune system’s role, particularly in AD, has gained considerable interest, with nanobodies representing a new frontier in biomedical research. Advances in targeting antibodies against amyloid-β (Aβ) and using messenger RNA for genetic translation have revolutionized the production of antibodies and drug development, opening new possibilities for treatment. Despite these advancements, conventional therapies for AD, such as Cognex, Exelon, Razadyne, and Aricept, often have limited long-term effectiveness, underscoring the need for innovative solutions. This necessity has led to the incorporation advanced technologies like artificial intelligence and machine learning into the drug discovery process for neurodegenerative diseases. These technologies help identify therapeutic targets and optimize lead compounds, offering a more effective approach to addressing the challenges of AD and similar conditions.

## 1. Introduction

The central nervous system (CNS) is the hub for intricate neurological processes, managing essential physiological functions, cognition, and motor activities. Various factors, including genetic predispositions and environmental exposures, contribute to CNS degradation. Neurodegenerative diseases such as Alzheimer’s disease (AD), Parkinson’s disease, and Huntington’s disease exemplify conditions where progressive neuronal damage leads to cognitive and motor dysfunction [1]. Apart from genetic factors, traumatic injuries, infections, and toxic exposures are significant contributors to CNS damage. This review aims to delve into the therapeutic potential within the molecular aspects of CNS degradation, emphasizing the need to enhance our understanding of these complexities to develop targeted interventions and explore neuroprotection strategies [2,3].

Neurodegenerative disorders cover a wide range of conditions characterized by the progressive loss and dysfunction of neurons, resulting in cognitive and motor impairments and, ultimately, severe disability.

The quest for a deeper understanding of neurodegenerative mechanisms and identifying new biomarkers and therapeutic targets is crucial in reducing their impact. Drug discovery in the context of neurodegenerative diseases is complex, demanding a deep knowledge of disease mechanisms, thorough testing, and innovative treatment strategies [4]. Recently, there has been a shift towards more creative and comprehensive approaches in drug discovery, utilizing advancements in genomics, proteomics, metabolomics, and computational biology. Techniques such as high-throughput screening, structure-based drug design, and drug repurposing have become invaluable in speeding up the identification and development of potential treatments. Furthermore, the integration of artificial intelligence (AI) and machine learning (ML) in drug discovery processes shows excellent potential in speeding up the identification of new drug targets, predicting drug effectiveness and safety, and refining drug candidates [5,6,7,8].

## 2. Alzheimer’s Disease (AD)

The deadliest neurodegenerative condition, where amyloid-β (Aβ) amyloidogenesis is believed to be the cause, is AD. Studies of its structure show that the fibrils composing Aβ (1–42) have disordered residues in positions 1–17, while residues 18–42 adopt a β-strand–turn–β-strand motif. This structure forms two intermolecular, parallel, in-register β-sheets, thanks to residues 18–26 (β1) and 31–42 (β2). Two Aβ (1–42) molecules form a repeating structure in a protofilament. The intermolecular side-chain interaction between odd-numbered residues of strand β1 from one molecule and even-numbered residues of strand β2 of the (n − 1)th molecule from another explains the sequence selectivity, cooperativity, and unidirectional growth of Aβ fibril growth. This interaction leads to partially unpaired β-strands at the fibril ends, contributing to amyloid plaque deposition in the brain, a critical factor in AD. Additionally, tau, a protein associated with microtubules, plays a significant role in AD by forming neurofibrillary tangles, another disease hallmark. Usually, tau helps maintain microtubule stability, but in AD, it becomes abnormally phosphorylated, causing microtubule disassembly and the formation of insoluble filaments that accumulate as neurofibrillary tangles in the brain [9,10]. Furthermore, glutamate-induced cytotoxicity, linked to mitochondrial dysfunction, oxidative stress, and autophagy activation, contributes to AD pathophysiology. Mitochondrial dysfunction, triggered by oxidative stress, has been noted in neuronal cells exposed to glutamate, underlining its link to excitotoxicity and its significance in AD. This cytotoxicity caused by glutamate is associated with an imbalance in mitochondrial dynamics, leading to hyperpolarization of mitochondria, increased production of reactive oxygen species (ROS), and heightened oxygen consumption. These factors contribute to mitochondrial dysfunction and oxidative stress, which are known to contribute to AD pathogenesis [11,12].

AD is a common neurological disorder leading to progressive dementia. Discovered roughly a century ago, its exact cause remains unknown. However, the accumulation of amyloid-β plaques and neurofibrillary tangles inside neurons are key markers of AD [13]. The most common Aβ peptides are Aβ40 and Aβ42, comprising 40 and 42 residues, respectively [14]. Consequently, various treatments targeting Aβ have been proposed for AD [15].

**Figure 1:**
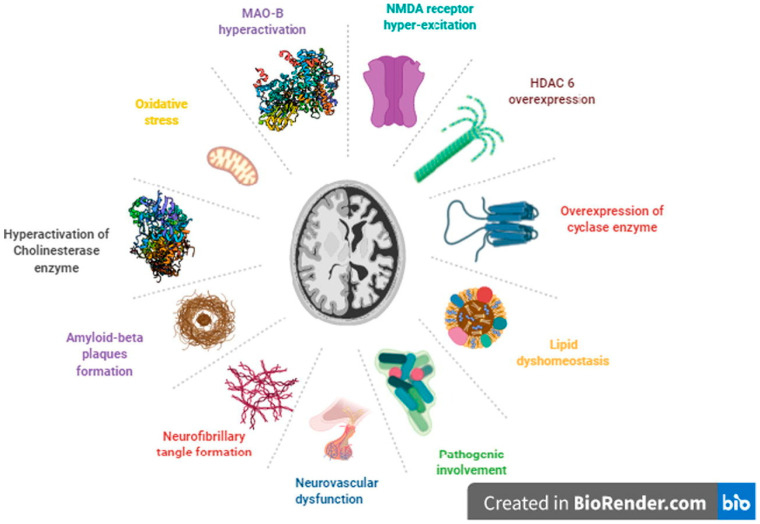
Key molecular mechanisms involved in the pathogenesis of Alzheimer’s disease [18].

The current therapeutic landscape for neurodegenerative disorders such as AD includes a wide range of interventions, from pharmacological agents to complementary and rehabilitative strategies. Pharmacotherapy with cholinesterase inhibitors, such as donepezil and rivastigmine, helps slow cognitive decline, while the NMDA receptor antagonist memantine addresses glutamatergic dysregulation [16]. Immunotherapeutic strategies targeting protein aggregates and modulating immune responses are being explored, marking a new direction in managing neurodegenerative diseases. This integrated approach reflects a comprehensive strategy to tackle the complex nature of AD, emphasizing the need for personalized and nuanced treatments to enhance efficacy and patient outcomes. Multi-target drug treatments are gaining attention as a strategic way to address neurodegenerative diseases [17] (Figure 1).

## 3. Multi-Target Drugs

The history of multi-target drug development has seen significant changes driven by a growing understanding of the complexity of neurodegenerative diseases [19]. Initially, drug development focused on single targets to identify and address key molecules or pathways. While successful in some cases, the limitations of this approach became clear, especially for complex diseases [20,21,22,23]. Advances in genomics and high-throughput technologies have led to a better understanding of diseases at the genetic and molecular levels. With the advent of systems pharmacology, the focus has shifted to multi-target drug development, treating neurodegenerative diseases as networks of interconnected pathways [24,25,26]. The future of this field may involve personalized medicine, leveraging individual genetic and molecular profiles, and exploring combination therapies for synergistic effects [27].

The pathogenesis of neurodegenerative disorders, such as AD, involves interconnected processes, including protein aggregation, oxidative stress, neuroinflammatory responses, and synaptic dysfunction [28]. Notably, memantine, an N-methyl-D-aspartate receptor antagonist, is the most recent AD treatment, approved over a decade ago [6,29]. Other standard therapies are rivastigmine, galantamine, and donepezil, which are cholinesterase inhibitors (ChEIs). Additionally, a combination therapy of memantine and donepezil, known as Namzaric, was approved in 2014 for treating individuals with moderate-to-severe AD who are stabilized on [30,31] both medications. This therapy combines two established agents in a fixed-dose product, offering an optimal approach for AD patients. Consequently, researchers are increasingly focusing on multi-target-directed ligand strategies to create hybrid molecules that can modulate multiple biological targets simultaneously [32,33] (Table 1).

### 3.1. Chemical-Based Drugs

Developing multi-target agents targeting related drug targets typically relies on two strategies. The first, the fragment-based method, involves combining pharmacophores from selective single-target ligands by linking or overlapping distinct pharmacophores based on structural similarities. However, linkers can lead to compounds with poor biopharmaceutical or pharmacokinetic profiles, such as those violating Lipinski’s rules. Although cleavable linkers present some advantages, they may also limit the benefits of multi-target approaches, such as simplified pharmacokinetics and reduced drug interactions. An alternative approach involves screening compound collections with multiple or single multi-tasking computational models to identify compounds with a desirable activity profile [34,35].

Recent studies have delved into multi-target drugs’ potential in addressing AD. A study published several years ago showed that simultaneously applying moderate inhibition to BACE1 and γ-secretase was effective and safe in Alzheimer’s mice models. This approach did not exhibit toxicity, unlike the serious side effects of inhibiting BACE1 or γ-secretase alone [36]. Similarly, the potential of acetylcholinesterase inhibitors (AChEis) in AD therapy has spurred the development of novel AChE-based multi-target drugs, especially those affecting Aβ production and tau phosphorylation without triggering AChE expression. AChE-based multi-target ligands were developed, including the lead compound M30 and its derivative M30D. M30D, incorporating essential elements from the FDA-approved anti-AD drug rivastigmine, functions as a prodrug of M30. It targets key disease-related enzymes and pathways in AD, such as Aβ, tau, AChE, monoamine oxidase A/B, metal dyshomeostasis, oxidative stress, and inflammatory and neuroprotective pathways [37,38,39]. Another study highlighted isoquinoline alkaloids from Zanthoxylum rigidum roots, particularly nitidine and avicine, for their multi-target activity. These compounds inhibit cholinesterase, monoamine oxidase A, and Aβ1–42 aggregation, which is crucial in AD pathology. The multi-target nature of nitidine and avicine suggests their potential as comprehensive therapeutic agents for AD [40]. Further research has focused on creating tetrahydroisoquinoline-benzimidazoles as versatile agents against AD. Some compounds in this group showed potent neuroinflammation and BACE1 inhibition, significant neuroprotective effects, and effective blood–brain barrier (BBB) penetration [41].

Additionally, cannabinoids, especially indazolylketones, have been recognized as promising multi-target agents for AD drug development. Researchers developed a new family of indazolylketones with a multi-target profile, including cholinesterase and BACE1 activities. After molecular docking and dynamics analysis, the most promising candidates were synthesized and experimentally tested, resulting in nine indazolylketones with significant multi-target activity [42,43]. In conclusion, the exploration of chemical-based multi-target agents for addressing Alzheimer’s disease holds promise, as evidenced by various compounds targeting key pathways and enzymes implicated in AD pathology, presenting a potential avenue for developing comprehensive therapeutic strategies.

### 3.2. Immune System-Modulating Drugs

The role of the immune system in neurodegenerative disorders, including AD, has gained significant attention, leading to the exploration of immune system-modulating drugs as potential treatments. The brain’s immune response, primarily mediated by microglia and astrocytes, is crucial for maintaining neuronal homeostasis, synaptic plasticity, and neuroprotection [44,45,46]. However, dysregulation in immune responses, chronic neuroinflammation, and accumulation of immune-related pathologies contribute to the progression of neurodegenerative diseases [47,48].

Several immune system-modulating strategies are under investigation for their potential to modulate neuroinflammatory processes, enhance neuronal survival, and mitigate disease progression. One strategy involves targeting specific immune receptors, such as the triggering receptor expressed on myeloid cells 2 (TREM2), which has been implicated in microglial activation and phagocytosis of amyloid beta aggregates in AD [49,50,51]. Modulating TREM2 signaling or enhancing microglial function through other immune receptors represents a promising therapeutic approach [51,52,53,54]. Additionally, cytokine-based therapies aimed at modulating pro-inflammatory and anti-inflammatory signaling pathways have been explored. For example, anti-inflammatory cytokines such as interleukin (IL)-10 may suppress neuroinflammation and promote neuro-regeneration.

Conversely, strategies aimed at inhibiting pro-inflammatory cytokines, such as tumor necrosis factor alpha and IL-1 beta (IL-1β), are being studied for their potential to reduce neuroinflammatory damage in neurodegenerative disorders [55,56,57]. Immunotherapeutic approaches that focus on clearing misfolded proteins, such as amyloid beta and tau, using monoclonal antibodies or active immunization, are exploring ways to modulate the immune system. These therapies leverage the immune system’s capacity to identify and eliminate pathological protein aggregates to reduce neuronal toxicity and slow disease progression.

While immune system modulation shows promise for treating neurodegenerative disorders, challenges remain in target specificity, potential side effects, and understanding immune–brain interactions. Ongoing research efforts utilizing advanced preclinical models, biomarker discovery, and rigorous clinical trials are vital to optimizing immune system-modulating drugs for AD and other neurodegenerative diseases. A significant advancement in this area is lecanemab (Leqembi), which was approved by the US Food and Drug Administration (FDA) for treating mild AD and mild cognitive impairment from AD [58]. This drug mimics naturally produced antibodies by the immune system. Research into calcineurin inhibitors and other pharmaceutical agents’ potential benefits in reducing dementia prevalence continues [59].

Investigations into fenamates, a class of non-steroidal anti-inflammatory drugs (NSAIDs), have shown their ability to target inflammation and improve memory deficits in animal models, highlighting the potential of immune system-focused interventions for AD and similar neurodegenerative disorders [60,61]. Moreover, immunotherapies such as daratumumab, which targets CD38, demonstrate potential for modulating the immune system in AD [62]. These findings underscore the growing interest in immune-modulating drugs as a promising strategy for treating neurodegenerative diseases.

Bapineuzumab, a monoclonal antibody aimed at amyloid beta—an essential protein in AD pathogenesis—enhances amyloid beta clearance from the brain and modulates immune responses. This dual action seeks to reduce neuroinflammation and the neurotoxicity associated with protein aggregation in AD [63,64]. Beyond specific antibodies like bapineuzumab, interest is increasing in multi-target therapeutic approaches that involve immune modulation. Combining antioxidants such as alpha tocopherol (vitamin E) with agents that affect glutamatergic neurotransmission aims to mitigate oxidative stress and excitotoxicity, which are known to cause neuronal damage in AD. Dietary supplementation with alpha tocopherol can help maintain glutathione levels and reduce oxidative stress markers in AD animal models. Vitamin E has also been found to protect against oxidative damage from beta amyloid and delay memory impairments.

Vitamin E, combined with selegiline, may slow the progression of moderate Alzheimer’s disease (AD), whether used alone or combined [65,66,67]. Additionally, enhancing neurotrophic factors, such as brain-derived neurotrophic factor (BDNF) and nerve growth factor (NGF), in combination with immune-modulating agents, represents a comprehensive approach to neurodegenerative disease treatment. BDNF, in particular, is more effective than NGF in restoring neural circuits, reducing cell loss, and improving neuronal function in AD [68,69,70,71]. This strategy aims to increase neuronal survival, enhance synaptic plasticity, and promote neuro-regeneration, potentially benefiting individuals with AD and other neurodegenerative disorders.

In conclusion, exploring immune system-modulating drugs represents a promising avenue for treating neurodegenerative disorders. Despite challenges in target specificity and understanding immune–brain interactions, ongoing research efforts continue to optimize these drugs, with recent advancements like lecanemab signaling potential breakthroughs. Combined with multi-target therapeutic approaches and strategies targeting specific immune receptors and cytokines, these interventions offer hope for mitigating neuroinflammation, reducing protein aggregation, and ultimately slowing disease progression.

### 3.3. Nanobodies

Nanobodies, a new class of antibody fragments derived from camelid antibodies, are gaining prominence in biomedical research. These fragments consist of a single variable domain from heavy-chain antibodies (VHH) and are the smallest antigen-binding fragments found naturally. Their diminutive size and high stability and solubility make them ideal for various therapeutic and diagnostic purposes [72], including Alzheimer’s disease [73,74]. Nanobodies are notable for their compact structure, typically comprising 110 amino acids. This minimalistic design endows nanobodies with several advantages, such as the ability to bind to cryptic epitopes inaccessible to conventional antibodies, high thermal stability, and ease of nanobody production in microbial systems. These features are particularly beneficial in targeting complex molecular structures including enzymes and toxins [75,76,77]. They offer advantages over traditional monoclonal antibodies, such as improved tissue penetration and the ability to be easily modified for therapeutic or diagnostic purposes due to their low immunogenicity and specificity for complex targets [78].

Research into nanobodies targeting amyloid-β (Aβ) plaques aims to develop targeted AD therapies and diagnostic tools. For example, a nanobody–drug conjugate that binds to Aβ plaques to deliver anti-Aβ drugs shows promise for targeted AD treatment [79]. Nanobodies are also being studied for their potential to modulate the immune system and reduce neuroinflammation, a characteristic of AD, which could improve cognitive function in AD models [80] (Table 2). To conclude, it is evident that nanobodies are emerging as a versatile tool in biomedical research and present exciting opportunities for advancing Alzheimer’s disease therapeutics and diagnostics. Their unique characteristics, including small size, high stability, and ability to target cryptic epitopes, offer distinct advantages over traditional antibodies. With ongoing research focusing on their application in targeting amyloid-β plaques and modulating immune responses, nanobodies hold promise for developing targeted therapies and improving understanding of AD pathology.

#### 3.3.1. Fab Fragments

Nanobodies, which may include Fab fragments, are essential due to their unique properties and potential in therapeutic and diagnostic applications. Derived from full-length antibodies, these fragments contain one constant variable domain of each heavy and light chain, crucial for retaining antigen binding specificity. Their smaller size offers advantages in tissue penetration and reduced immunogenicity, making them suitable for targeted applications. These fragments can bind to antigens with high specificity, a property crucial for their use in various medical applications [81]. In therapeutics, their reduced size facilitates enhanced tissue penetration, allowing for effective targeting and drug delivery to specific cells or tissues, including tumor cells [82].

F(ab’)2 fragments, possessing two antigen binding sites, bind antigens with increased avidity compared to monovalent Fab fragments. This bivalency is vital for certain therapeutic and diagnostic applications where antigen cross-linking is essential. Despite being larger than Fab fragments, F(ab’)2 fragments offer improved tissue penetration and reduced immunogenicity due to the absence of the Fc region [83]. They are typically generated through enzymatic digestion of antibodies with pepsin. This cleaves the antibody molecule below the hinge region, leaving the F(ab’)2 portion intact, yielding fragments with two antigen binding sites for various applications [81].

The anti-Aβ antibody 3D6 (bapineuzumab) Fab fragment and other anti-Aβ antibody Fab fragments can effectively inhibit Aβ deposition and reduce neurotoxicity in a young AD mouse model [84]. Similarly, the recombinant Fab antibody 1E8-4b targets AD-related Aβ peptides, namely, Aβ(1–40), Aβ(1–42), and Aβ(1–43). Subsequently, the binding of the 1E8-4b recombinant Fab antibody to plaques in brain tissues obtained from CERAD-defined AD patients was demonstrated through immunohistochemistry [85].

Evidently, fab fragments offer significant potential in therapeutic and diagnostic contexts due to their unique attributes. These fragments, derived from full-length antibodies, retain antigen-binding specificity while benefiting from reduced size and enhanced tissue penetration. Furthermore, Fab fragments like the anti-Aβ antibody 3D6 (bapineuzumab) and recombinant Fab antibody 1E8-4b demonstrate promising efficacy in inhibiting Aβ deposition and targeting AD-related peptides, highlighting their relevance in neurodegenerative disease research.

#### 3.3.2. Domain Antibodies

Domain antibodies (dAbs) are emerging as a novel class of therapeutic and diagnostic tools in biotechnology. They are the smallest functional units derived from antibodies, containing a single variable domain from conventional antibodies’ heavy (VH) or light (VL) chains. Their minimalistic structure offers several advantages for biomedical uses. The key attribute of domain antibodies is their compact size, which allows for high stability, straightforward production in various systems, and the ability to target areas inaccessible to larger antibodies. Their simple structure also makes them suitable for engineered modifications for specific applications [86]. Single-domain antibodies can identify and bind selectively to Aβ oligomers in vitro, inhibit fibril formation, and prevent Aβ-induced neurotoxicity [87]. dAbs targeting tau protein aggregates have been investigated for their potential to halt their formation and spread, a critical aspect of AD. For instance, a dAb that binds to and inhibits tau aggregation has shown promise in reducing neurodegeneration in AD models. Studies have also investigated dAbs against inflammatory cytokines, showing neuroprotective effects in AD models and hence hold the potential to further the multi-target drug discovery [9,88].

#### 3.3.3. Single-Chain Variable Fragments (scFv)

Single-chain variable fragments (scFvs) significantly advance antibody engineering, linking the variable regions of an antibody’s heavy and light chains with a short peptide. This design retains antigen binding capability while offering benefits in size and modifiability [89]. scFvs are small and flexible, allowing for them to bind to epitopes that are inaccessible to more significant antibodies. The single-chain design reduces the likelihood of mispairing with endogenous immunoglobulin chains, leading to improved specificity and reduced immunogenicity. Additionally, scFvs can be engineered to increase their affinity and stability, which makes them suitable tools for various applications [90].

Research into scFv antibodies for AD treatment has included the development of elongated scFv mutants of the antibody bapineuzumab, showing potential for efficient treatment at low dosages, potentially avoiding adverse side effects associated with the parent monoclonal antibody (mAb). The elongated scFv mutants were designed to drive Aβ1–42 oligomers to the non-toxic pathway, offering a new approach to AD treatment. Additionally, scFv antibodies have been explored for their ability to disaggregate amyloid-β-42, indicating their potential as immunotherapeutic agents for AD. Moreover, anti-Aβ scFv antibodies exert synergistic neuroprotective activities in AD models, highlighting their potential in restoring memory acquisition in the context of AD [91,92].

### 3.4. Antibody Targeting

Targeting antibodies against amyloid beta (Aβ) effectively tackles the amyloid cascade issue in AD. Aducanumab, a monoclonal antibody targeting the fibrillar form of Aβ in the brain, has been approved for AD treatment. Lecanemab targets prefibrillar Aβ species, and small-molecule inhibitors such as COR 388 are proposed as potential treatments for amyloid-β [93,94]. Despite amyloid proteins being self-assembly units that catalyze their aggregation, the complexity of Aβ aggregation makes it challenging to target with antibodies and molecular receptors, thus redirecting the peptide away from the fibrillation process and necessitating alternative treatment strategies. Recent efforts have focused on creating mutants of pathological Aβ structures to inhibit kinetically amyloid aggregation [95]. However, introducing new antigens may lead to undesirable off-target effects.

**Figure 2:**
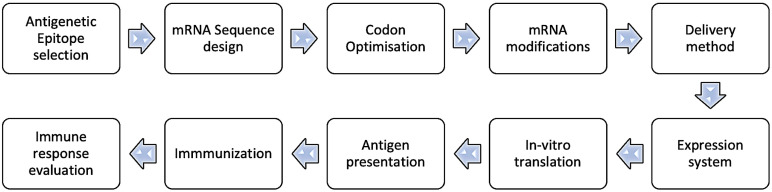
Aβ42 modification for immunogenicity without direct logging.

Developing antibodies for Aβ40 or Aβ42 depends on the specificity of these forms, with Aβ42 being more hydrophobic and primarily responsible for aggregates [96]—specific and compelling reduction of these aggregates if an antibody is generated to target Aβ42 specifically. However, the protein translated is not the same as Aβ42, leading to adverse effects when the same approach is applied. The successful transition of a peptide to the antigenic state in mRNA translation depends on various factors, including the choice of epitope, the type of host organism, and the method of the antigenic peptide delivery. To ensure immunogenicity without direct logging, the Aβ42 should be modified in a stepwise manner (Figure 2):

1. Select an Antigenic Epitope: Identify a specific sequence or epitope known to be antigenic, crucial for antibody–antigen interaction in AD.
2. Design mRNA Sequence: Create an mRNA sequence encoding the chosen epitope, incorporating a 5′ cap and a 3′ poly-A tail to align with transcription, such as starting with a 5′ cap and including a 3′ poly-A tail. Ensure the sequence is in-frame with the ribosome so that translation produces the desired epitope.
3. Codon Optimization: Optimize the mRNA sequence for effective translation in the desired host cell, mainly by selecting codons frequently used by the host.
4. Consider mRNA Modifications: To boost stability and translation, integrate modified nucleotides such as pseudouridine or 5-methylcytidine into the mRNA sequence, which minimizes immune recognition. Alternatively, the replacement of uridine with pseudouridine is also a practical approach.
5. Delivery Method: Decide on the delivery approach for the mRNA to the target cells, such as electroporation, lipid nanoparticles, or viral vectors.
6. Expression System: Select an efficient expression system for effective mRNA translation and epitope production, such as a suitable cell line or organism.
7. In Vitro Translation: Verify the mRNA’s ability to produce the desired epitope through in vitro translation systems.
8. Antigen Presentation: Process and present the translated antigenic peptide on the cell surface via major histocompatibility complex (MHC) molecules for immune recognition.
9. Immunization: Use the peptide to immunize, stimulating an immune response as part of a vaccine or immunotherapy.
10. Immune Response Evaluation: Assess the immune response by measuring antibody or T-cell reactions against the peptide using enzyme-linked immunosorbent assay (ELISA), flow cytometry, or cytokine assays.

Matching the Fc region of an antibody with an epitope sequence combines bioinformatics and molecular biology techniques. Understanding the unique functions and structures of the Fc region and epitope is crucial. The Fc region [97] connects with cell surface receptors and complement proteins at an epitope, the specific part of an antigen recognized by the immune system [98].

The next step involves retrieving and aligning these sequences using tools and databases such as GenBank and BLAST [99,100]. When 3D structures are unclear, homology modeling tools are essential for predicting these structures. Software such as PyMOL 2.6 and UCSF Chimera X allows for researchers to visualize and analyze these structures in depth [101,102].

The mystery of the interaction between the Fc region and the epitope starts to clear up during docking studies. Tools such as HADDOCK [103] and ClusPro [104] simulate these complex interactions, showing binding affinities and interaction sites. However, experimental validation is crucial. Techniques such as site-directed mutagenesis and binding assays (e.g., ELISA, surface plasmon resonance) provide concrete data that support the computational findings.

Statistical tools are vital in interpreting the data, identifying significant patterns and insights, and leading to solid conclusions. Further, in vivo studies enhance these findings, offering a detailed understanding of Fc–epitope interactions within a biological context.

### 3.5. mRNA-Based Antibodies

**Figure 3:**
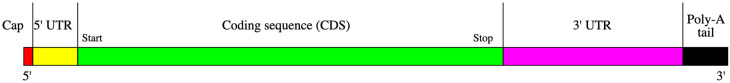
mRNA sequence of the Aβ42.

Messenger RNA (mRNA) is crucial in converting genetic information from DNA into proteins, comprising several essential components [105,106]. An mRNA sequence can be designed for an antibody targeting the Aβ42 with 42 amino acids, illustrating its two epitopes (Figure 3, Table 3 and Table 4).

Reducing Aβ42 in the brain is expected to affect Alzheimer’s disease (AD) status significantly. The structure–toxicity relationship shows that Aβ amyloid fibrils’ intrinsic toxicity correlates with their morphology (e.g., more regular, and extended fibrils tend to be more toxic). This observation, which is in agreement with previous observations on various aggregation stages of Aβ(1–42) [107], supports the idea that neuronal degeneration in AD is due to Aβ amyloid deposition [108]. However, oligomers resembling small diffusible ligands (ADDLs, or Aβ-derived diffusible ligands) [109] or protofibrils [110] are toxic and may play a key role in AD. These oligomeric structures share common structural features different from amyloid fibrils [111].

The structure of Aβ (1–42) protofilament provides insights into the selectivity, cooperativity, and directionality of Aβ fibril growth. It also offers a structural basis for the mechanism of current Aβ fibrillization inhibitors and shows a link between neurotoxicity and different Aβ conformations, potentially aiding in understanding amyloidogenesis of AD and the development of effective anti-AD drugs and diagnostic markers.

Traditional methods of multi-target drug discovery have relied on fragment-based approaches, combining pharmacophores from selective single-target ligands to design compounds with desired multi-target activity of various types, as discussed above. While effective, these methods often face challenges such as linker-associated issues and limited biopharmaceutical profiles. However, recent advancements in AI and machine learning (ML) have revolutionized the field by enabling the rapid analysis of vast molecular datasets and predicting compound activity profiles with unprecedented accuracy. AI-driven approaches facilitate target identification and repurposing of existing drugs for complex diseases like Alzheimer’s. By harnessing the power of AI, researchers can expedite the discovery of multi-target drugs, overcoming traditional limitations and offering new avenues for therapeutic intervention in neurodegenerative disorders, as discussed in the following section.

## 4. AI-Driven Multi-Target Drugs

AI and ML are increasingly used in drug discovery for neurodegenerative diseases such as AD. These technologies assist in target identification, lead generation, and optimization, speeding up the development of new treatments. A key focus is repurposing existing drugs for these disorders. AI and ML-based frameworks have shown promise in identifying potential candidates for repurposing, especially for complex conditions such as AD, by analyzing vast amounts of molecular, structural, and clinical data to find new therapeutic options and combination therapies for neurodegenerative diseases.

Combining various data sources, such as genomics, bioinformatics, and clinical data, enhances the integration of AI and ML in drug repositioning for neurodegenerative diseases. This method is promising for speeding up the discovery of new therapeutic options and improving the success rate of drug development for neurodegenerative disorders. A notable example is the identification of novel drug targets for neurodegenerative disorders, notably AD, using the AI-driven PandaOmics tool in silico and the FuzDrop methodology developed at the University of Cambridge. This approach led to the discovery of proteins involved in phase separation dysregulation, which is critical to disease pathology. By analyzing human sample data, the researchers assessed the role of protein phase separation in disease-related processes. High-ranking candidates identified by PandaOmics and FuzDrop were prioritized, leading to the discovery of potential therapeutic targets related to phase separation in diseases. Experimental validation in Alzheimer’s disease cell models confirmed the involvement of three predicted targets (MARCKS, CAMKK2, and p62), validating their roles in Alzheimer’s and underscoring their potential as therapeutic intervention points, suggesting that interventions targeting these proteins could potentially counteract the pathological processes underpinning AD [112].

Similarly, researchers at the University of Arizona College of Medicine used AI to explore the molecular changes in healthy neurons during the progression of AD, identifying complex pathways involved in the disease. This AI and big data-driven approach highlights the potential for developing innovative treatments by targeting newly identified or combination pathways in AD [113]. Additionally, a Bayesian ML model utilizing data from ChEMBL and PubChem databases aimed to identify a novel small molecule with therapeutic potential for Alzheimer’s, leading to the identification of GSK3β as a promising target [114]. Galantamine, initially developed for poliomyelitis treatment, was repurposed, and approved by the US FDA for AD treatment.

Similarly, drugs such as fluoxetine and levetiracetam, known, respectively, for serotonin reuptake inhibition and antiepileptic properties, have shown efficacy in AD management. Network pharmacology and data from the ChEMBL database have been instrumental in establishing a comprehensive drug–protein interaction network specific to AD and identifying three multi-target drugs approved for AD (rivastigmine, memantine, and donepezil) and five single-target medications (aducanumab, florbetapir, galantamine, florbetaben, and flumetamol) endorsed for AD treatment [115,116].

Evidently, AI and ML have revolutionized drug discovery. However, optimizing delivery mechanisms for multi-target drugs remains a crucial challenge to ensure efficacy in treating complex diseases like Alzheimer’s. The blood–brain barrier (BBB) poses a formidable challenge to effective medication delivery to the central nervous system (CNS), rendering brain disorders like Alzheimer’s difficult to treat.

## 5. Drug Delivery across the BBB

A significant barrier to effective medication delivery to the CNS is the BBB, making many brain disorders incurable. Typically, only small-molecule drugs can penetrate the BBB, leading to a prolonged focus on developing, testing, and refining such compounds that act at specific brain sites. However, small-molecule drugs face challenges such as non-specific targeting, widespread organ distribution, low therapeutic indices, rapid development of drug resistance after treatment initiation, crossing of the BBB by fewer small-molecule drugs, and minimal CNS activity (i.e., only 1% of all drugs exert action in the CNS) [117].

A limited number of brain disorders, such as epilepsy, chronic pain, and depression, respond to small-molecule drugs. However, most severe brain conditions, including Huntington’s disease, multiple sclerosis, AD, Parkinson’s disease, brain cancer, stroke, brain and spinal cord injury, HIV infection, and numerous childhood inborn genetic errors affecting the brain, involve poor response to these treatments. Some FDA-approved small-molecule drugs, such as levodopa for Parkinson’s disease and Cognex, Exelon, Razadyne, and Ariecept for AD, offer temporary symptom relief but eventually lose effectiveness.

Due to their typically low BBB permeability, developing large-molecule medicines is often discouraged. Many promising large-molecule drugs, effective in ex vivo studies, have not advanced to clinical use because they cannot sufficiently penetrate the CNS. This category includes engineered proteins (e.g., nerve growth factors), antibodies, genes, vectors, micro-RNA, siRNA, oligonucleotides, and ribozymes.

The BBB is formed by tight junctions resulting from the interaction of several transmembrane proteins that close off paracellular pathways. This complex network, particularly involving occludin and claudin proteins, effectively blocks polar solutes in blood from freely diffusing along potential paracellular channels in water, thereby preventing their entry into the cerebrospinal fluid. Despite significant scientific efforts, the challenge to traverse the BBB persists, with developed methods such as liposome use or charged lipid formulations, which have limited complex stability in serum and high toxicity over time [118]; electroporation-based techniques, which are temporally limited and risk bioactivity loss [119]; and viral-based vectors and fusions, facing safety and efficacy concerns. Typically, achieving targeted delivery necessitates invasive procedures, such as direct brain injection [120], underscoring the ongoing need for innovative BBB penetration solutions.

### Strategies That Aid Drugs Cross the Blood–Brain Barrier

1. Invasive techniques include intra-cerebral injection, convection-enhanced delivery, and intra-cerebroventricular infusion [121].
2. BBB disruption with bradykinin analogs, ultrasonography, and osmotic pressure [122].
3. Physiological procedures involving transporter-mediated delivery, receptor-mediated transcytosis, and adsorptive-mediated transcytosis [123].
4. Pharmacological techniques involving liposome-mediated drug delivery or chemically modifying pharmaceuticals to lipophilic molecules [124].
5. Opsonization and drug delivery by nanoparticles across the BBB, wherein the drug is adsorbed onto the particles passively [125].

Although nanoparticles have significantly advanced medical science, most research has involved medications not covalently bonded with nanoparticles, potentially hindering nanomedicine from achieving its full potential.

Antibodies can specifically target receptors on the endothelial cells lining the BBB, facilitating receptor-mediated transcytosis. This method involves antibodies binding to these receptors, initiating an internal process that transports them and their drug cargo across the BBB. The efficacy of antibody-based drug delivery can be enhanced by decreasing their affinity for a transcytosis target, thereby increasing their brain uptake [126]. Bispecific antibodies designed to target the transferrin receptor for BBB crossing and an amyloid beta peptide to mitigate its accumulation in AD showcase the potential of engineered antibodies for dual targeting and therapeutic benefits [127].

## 6. Conclusions

The complexity of neurodegenerative diseases calls for innovative therapeutic strategies that address the multifaceted nature of CNS degradation. Multi-target drug interventions are a promising approach for tackling Alzheimer’s disease by simultaneously targeting multiple pathological mechanisms. Integrating AI/ML technologies into drug discovery accelerates the identification of potential therapeutic strategies for these disorders. Researchers can break through traditional drug development barriers by leveraging computational methods, predictive modeling, and data-driven insights, paving the way for precision medicine in neurodegenerative disorders. However, this approach faces several limitations and challenges, including the need for a deep understanding of disease mechanisms, identifying and validating suitable drug targets, and requiring substantial computational resources and data infrastructure. Ethical, data privacy, and regulatory considerations, and the heterogeneity of patient populations and disease progression further complicate drug development and clinical implementation. Despite these challenges, the collaborative efforts in multi-target drug discovery and AI-driven approaches are promising for advancing neurodegenerative therapeutics. Although it may take decades for mRNA vaccine treatments to reach patients, US government funding initiatives for novel biotechnology research [128] could accelerate their availability. Given the vast potential of this technology and its humanitarian impact, many new multi-target drug treatments are expected to arrive soon.

## Figures and Tables

**Table 1:** Multi-target drugs for Alzheimer’s disease and their functions.

Drug Name	Target(s)	Function(s)	Stage of Development
Aβ oligomer inhibitors (e.g., *BAN2401*, *aducanumab*)	Amyloid-β oligomers	Prevent or disassemble toxic clumps of amyloid-β	Clinical trials (aducanumab recently received FDA approval)
BACE1 inhibitors (e.g., *verubecestat*, *MK-8931*)	β-Secretase 1 (BACE1)	Reduce production of amyloid-β by inhibiting the enzyme that cleaves its precursor	Clinical trials (some promising results, others halted due to lack of efficacy)
Tau aggregates inhibitors (e.g., *P-tau217 PET tracers*, *LMTX*)	Tau protein aggregates	Prevent or remove tangles of misfolded tau protein	Preclinical/early clinical trials (imaging agents more advanced than therapeutic agents)
Cholinesterase inhibitors (e.g., *donepezil*, *rivastigmine*, *galantamine*)	Acetylcholinesterase (AChE)	Increase levels of the neurotransmitter acetylcholine, which is depleted in AD	Approved for symptomatic treatment of mild-to-moderate AD
NMDA receptor modulators (e.g., *memantine*)	N-methyl-D-aspartate (NMDA) receptors	Protect neurons from excitotoxicity and improve cognitive function	Approved for moderate-to-severe AD
Multi-target drugs (e.g., *J147*, *AV-1750*, *CTS-5559*)	Combinations of targets from above (e.g., AChE + NMDA, BACE1 + tau)	Address multiple aspects of AD pathology for potentially greater efficacy	Preclinical/early clinical trials (potentially more effective but require careful design and validation)

AChE: acetylcholinesterase; AD: Alzheimer’s disease; FDA: Food and Drug Administration; PET: positron emission tomography.

**Table 2:** Nanobodies in Alzheimer’s disease treatment: a summary.

Type of Nanobodies	Description	Mechanism of Action	Advantage	Disadvantage
Fab fragments	Modified antigen-binding fragments of conventional antibodies	Bind to specific targets, trigger immune response	High affinity, good specificity	Large size, limited tissue penetration
Domain antibodies	Single variable domains from antibodies with only the heavy chain (VH)	Bind to specific targets, inhibit specific pathways	Smaller than Fab fragments, they have potentially better tissue penetration	Less potent than Fab fragments, limited repertoire
Single-chain variable fragments (scFv)	Engineered fusion of heavy and light chain variable domains	Bind to specific targets, can be engineered for additional functions	Smaller than Fab fragments, customizable	Lower affinity than Fab fragments, limited potential stability

**Table 3:** Description of the proposed vaccine structure for Aβ42 with base modifications (mRNA sequence) (uridine is replaced with Ψ = 1-methyl-3′-pseudouridylyl).

Element	Description	Position
cap	A modified 5′-cap 1 structure (m7G+m3′-5′-ppp-5′-Am)	1–2
5′-UTR	The 5′-untranslated region derived from human alpha globin RNA with an optimized Kozak sequence.	3–54
sig	S glycoprotein signal peptide (extended leader sequence) guides translocation of the nascent polypeptide chain into the endoplasmic reticulum.	55–102
ORF	Codon-optimized sequence: GAAΨΨ ΨCGCC AΨGAΨ AGCGG CΨAΨG AAGΨG CAΨCA ΨGGCA GCGGC AGCGG CAGCG GCAGC GAGAΨ GΨGG GCAGC AACAA AGGC	103–187
3′-UTR	The 3′ untranslated region comprises two sequence elements derived from the amino-terminal enhancer of split (AES) mRNA and the mitochondrial encoded 12S ribosomal RNA to confer RNA stability and high total protein expression: GCΨAG CΨGCC CCΨΨΨ CCCGΨ CCΨGG GΨACC CCGAG ΨCΨCC CCCGA CCΨCG GGΨCC CAGGΨ AΨGC ΨCCCA CCΨCC ACCΨG CCCCA CΨCAC CACCΨ CΨGCΨ AGΨΨC CAGAC ACCΨCC CAAGC ACGCA GCAAΨ GCAGC ΨCAAA ACGCΨ ΨAGCC ΨAGCC ACACC CCCAC GGGAA ACAGC AGΨGA ΨΨAAC CΨΨΨA GCAAΨ AAACG AAAGΨ ΨΨAAC ΨAAGC ΨAΨAC ΨAACC CCAGG GΨΨGG ΨCAAΨ ΨΨCGΨ GCCAG CCACA CCCΨG GAGCΨ AGC	188–456
poly(A)	A 110-nucleotide poly(A)-tail consisting of a stretch of 30 adenosine residues, followed by a 10-nucleotide linker sequence and another 70 adenosine residues: AAAAA AAAAA AAAAA AAAAA AAAAA AAAAA GCAΨA ΨGACΨ AAAAA AAAAA AAAAA AAAAA AAAAA AAAAA AAAAAA AAAAA AAAAA AAAAA AAAAA AAAAA AAAAA AAAA	457–566

**Table 4:** Target Antigen of Aβ42 with its sequence and identified epitopes that can be linked via the (GS)4 linker as shown in red.

**Target Antigen:** Aβ42	DAEFRHDSGYEVHHQKLVFFAEDVGSNKGAIIGLMVGGVVIA					42 amino acids
**Linked epitopes:** (http://tools.iedb.org/bcell/)	No.	Start	End	Peptide	Length	EFRHDSGYEVHH -GSGSGSGS- EDVGSNKG
	1	3	14	EFRHDSGYEVHH	12	
	2	22	29	EDVGSNKG	8	
